# Low-Grade Appendiceal Mucinous Neoplasm Involving the Endometrium and Presenting with Mucinous Vaginal Discharge

**DOI:** 10.1155/2016/6841989

**Published:** 2016-10-23

**Authors:** Vera Vavinskaya, Joel M. Baumgartner, Albert Ko, Cheryl C. Saenz, Mark A. Valasek

**Affiliations:** ^1^Department of Pathology, University of California San Diego, 200 W. Arbor Drive MC 8320, San Diego, CA 92103, USA; ^2^Department of Surgery, University of California San Diego, Moores Cancer Center, 3855 Health Sciences Drive, La Jolla, CA 92093, USA; ^3^Kaiser Permanente, 10800 Magnolia Ave., Riverside, CA 92505, USA; ^4^Department of Reproductive Medicine, University of California San Diego, Moores Cancer Center, 3855 Health Sciences Drive, La Jolla, CA 92093, USA

## Abstract

Primary appendiceal mucinous lesions are uncommon and represent a spectrum from nonneoplastic mucous retention cysts to invasive adenocarcinoma. Low-grade appendiceal mucinous neoplasms (LAMNs) represent an intermediate category on this spectrum and can be classified according to whether or not they are confined to the appendix. Although LAMNs are frequently confined to the appendix, they can also spread to the peritoneum and clinically progress as pseudomyxoma peritonei (i.e., mucinous ascites). Thus, the appropriate classification of appendiceal primary neoplasia is essential for prognosis and influences clinical management. In addition, the precise classification, management, and clinical outcome of patients with disseminated peritoneal disease remain controversial. Here, we report an unusual case of LAMN with pseudomyxoma peritonei that initially presented with mucinous and bloody vaginal discharge. Pathological evaluation revealed low-grade appendiceal mucinous neoplasm with secondary involvement of the peritoneum, ovaries, and endometrial surface. Therefore, LAMN should be considered in the differential diagnosis of mucinous vaginal discharge.

## 1. Introduction

Primary neoplasms of the appendix are found in less than 2% of surgically resected appendices [1]. Despite the relatively low incidence, appendiceal lesions account for the vast majority of the pseudomyxoma peritonei (i.e., mucinous ascites), even in women with concomitant ovarian mucinous tumors [2]. Ovarian origin for mucinous ascites can rarely occur, generally in the context of mucinous adenocarcinoma arising from a mature teratoma [3, 4]. The classification of appendiceal mucinous tumors is controversial and terminology can be inconsistent, in particular, when there is lack of overtly malignant features; however, the accepted terminology for low-grade neoplasms without overt features of adenocarcinoma is LAMN [5]. Importantly, LAMN is nevertheless associated with extra-appendiceal spread as pseudomyxoma peritonei [6]. The nature and the grading of pseudomyxoma peritonei itself have also been controversial.

Approximately 25% of appendiceal mucinous neoplasms are clinically asymptomatic and found incidentally either on abdominal imaging or during surgery [7]. Occasionally these tumors may present with intestinal obstruction, intussusceptions, gastrointestinal bleeding, and external ureteral compression. We report a case of a mucinous appendiceal neoplasm that presented with mucinous/bloody vaginal discharge.

## 2. Case Report

A 41-year-old female with no significant past medical history presented to her primary care physician with mucinous and bloody vaginal discharge. A pelvic ultrasound revealed thickened endometrium. An endometrial biopsy was performed and demonstrated mucinous epithelium of gastrointestinal origin. The patient underwent a colonoscopy and an esophagogastroduodenoscopy, which revealed no evidence of a primary tumor. She then underwent a diagnostic laparoscopy which showed diffuse peritoneal carcinomatosis of the diaphragm, anterior surface of the liver, omentum, pelvic peritoneum, and a mucinous mass lesion in the distal appendix. Initially, an appendectomy was performed. Slides from the appendectomy specimen were reviewed and demonstrated fibrous obliteration of the majority of the appendiceal lumen with a low-grade appendiceal mucinous neoplasm present at the tip of the appendix. No destructive desmoplastic invasion was identified. There was an area of possible microscopic perforation with rare strips of extra-appendiceal neoplastic epithelium and the final pathologic diagnosis was LAMN with a high risk of recurrence.

**Figure 1:**
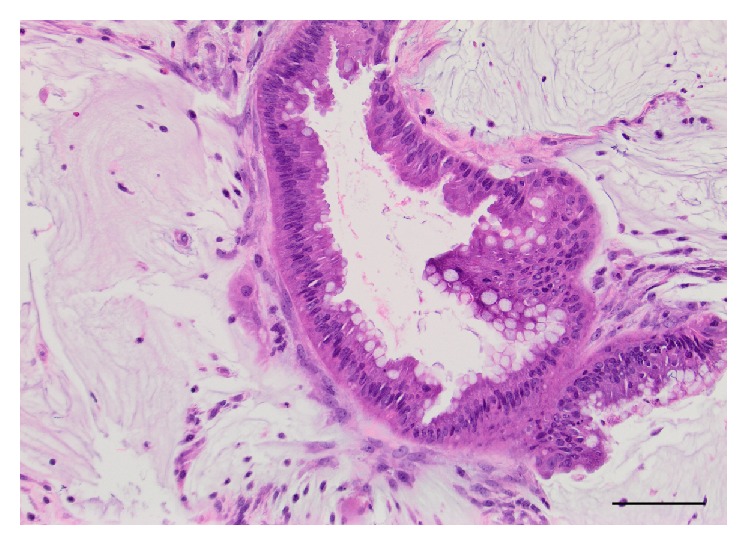
Peritoneal biopsies demonstrating involvement by low-grade appendiceal mucinous neoplasm (i.e., “low-grade mucinous carcinoma peritonei”); 20x, H&E, and scale bar = 100 micrometers.

**Figure 2:**
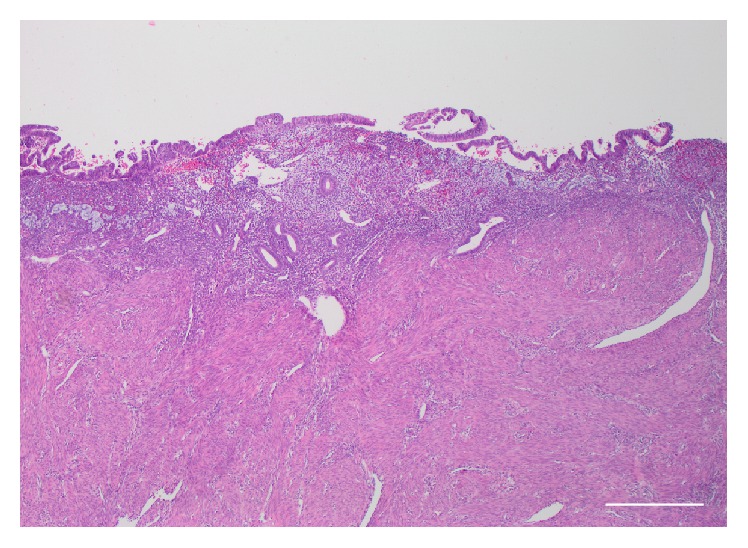
Diffuse reepithelialization of endometrium by low-grade appendiceal mucinous neoplasm with underlying weakly proliferative endometrium with focal hemorrhage; 4x, H&E, and scale bar = 500 micrometers.

**Figure 3:**
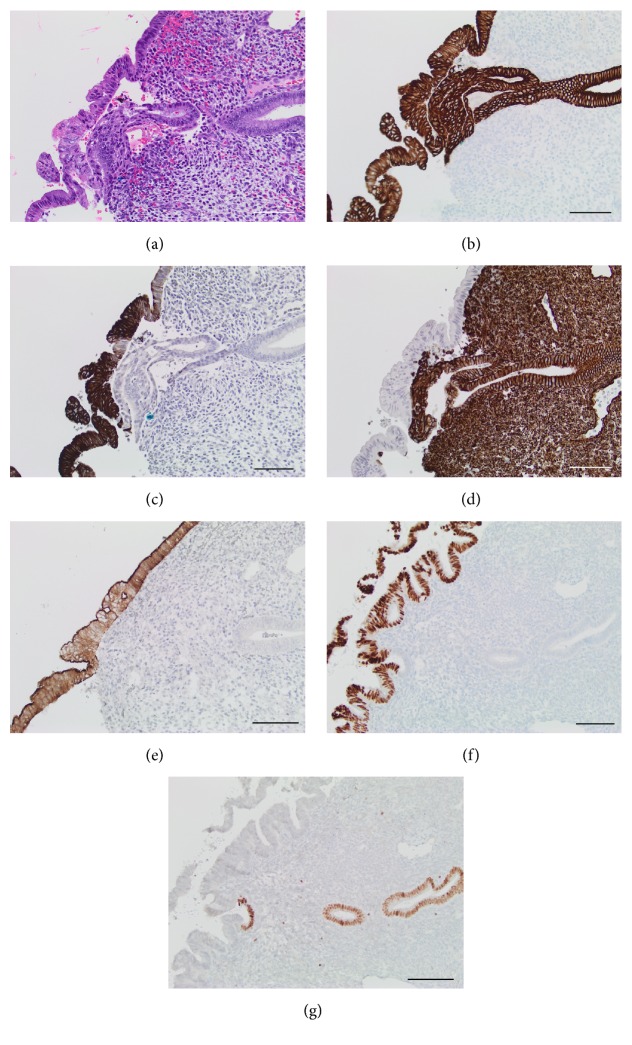
(a) High power view of the endometrium with the somewhat more eosinophilic neoplastic surface epithelium as compared to the underlying nonneoplastic endometrial glands; 20x, H&E, and scale bar = 100 micrometers. (b) Strong immunoreactivity for CK7 in both neoplastic surface epithelium and nonneoplastic endometrial glands; 20x and scale bar = 100 micrometers. (c) Strong immunoreactivity for CK20 in the neoplastic surface epithelium but not in nonneoplastic endometrial glands; 20x and scale bar = 100 micrometers. (d) Strong immunoreactivity for vimentin in nonneoplastic endometrial glands and stroma but not in neoplastic surface epithelium; 20x and scale bar = 100 micrometers. (e) Strong immunoreactivity for villin in the neoplastic surface epithelium but not in nonneoplastic endometrial glands; 20x and scale bar = 100 micrometers. (f) Strong immunoreactivity for Cdx-2 in the neoplastic surface epithelium but not in nonneoplastic endometrial glands; 20x and scale bar = 100 micrometers. (g) Strong immunoreactivity for Pax-8 in nonneoplastic endometrial glands but not in neoplastic surface epithelium; 20x and scale bar = 100 micrometers.

She was referred to our institution for further surgical therapy where she underwent complete cytoreductive surgery with hyperthermic intraperitoneal chemotherapy. This included omentectomy, excision of mucin from the lesser sac and bowel surfaces, peritonectomy of the bilateral diaphragms and pelvis, hysterectomy, and bilateral salpingo-oophorectomy. Pathology demonstrated low-grade mucinous neoplasm diffusely involving the endometrial surface, ovaries, and other resected specimens. The neoplastic endometrial surface epithelium was histomorphologically identical in appearance to the peritoneal disease (Figures 1 and 2). There was no invasion of endometrial stroma. Immunohistochemical stains demonstrated that the neoplastic epithelium was positive for CK7, CK20, villin, and CDX-2 and negative for vimentin and PAX-8, indicating intestinal differentiation (Figures 3(a)–3(g)). All immunohistochemical studies had appropriately positive external and internal tissue control staining. The patient continues to do well and is without evidence of recurrence 2 years after surgery.

## 3. Discussion

Tumors of the appendix are uncommon entities that comprise less than 2% of all appendectomies with an approximately 0.3% reported prevalence of mucinous neoplasms in the appendectomy specimens [8]. In the clinical setting of mucinous ascites or when diagnosing a primary appendiceal mucinous neoplasm, it is crucial to establish an accurate diagnosis as it dramatically influences the subsequent clinical decisions [2]. As seen in this case, the initial approach of immunohistochemical panel of CK7 and CK20 aids the pathological confirmation of endometrial involvement. In addition, diffusely positive immunostaining for CDX-2, a transcription factor involved in the embryologic development of alimentary structures, and villin, a marker for intestinal microvillous structures, supports gastrointestinal differentiation [2]. Although the histological differential diagnosis includes mucinous/intestinal metaplasia with or without associated intraepithelial neoplasia or endometrial carcinoma, there was no evidence of associated tubal features, ciliated epithelium, reserve cells, other forms of metaplasia (i.e., squamous metaplasia), hyperplasia, architectural complexity, or endometrial or cervical neoplasia. The mild cytologic atypia observed was restricted to the surface epithelium. Given the diffuse nature of the process, the histomorphological similarity to the peritoneal disease, the strongly positive gastrointestinal markers, and the completely negative endometrial markers, the findings are consistent with derivation from the known appendiceal primary and extensive peritoneal disease in this patient.

Therefore, this case further establishes the capacity of appendiceal primary neoplasia to colonize the endometrial surface (presumably via transit through the fallopian tubes), as both acellular mucin and neoplastic mucinous epithelium have previously rarely been observed to involve the endometrial surface in the context of an appendiceal mucinous neoplasm with pseudomyxoma peritonei [9–11].

The classification of appendiceal mucinous tumors is controversial. One of the pathologic diagnostic difficulties is appropriately distinguishing LAMN from mucinous adenocarcinoma of the appendix. According to Misdraji et al., appendiceal mucinous tumors with destructive invasion of the appendiceal wall, complex epithelial proliferations, or high-grade nuclear cytology generally pursue an aggressive clinical course and should be classified as mucinous adenocarcinomas [12]. In our case there was no evidence of destructive desmoplastic invasion or high-grade cytologic features, and therefore the tumor was classified as LAMN with low-grade pseudomyxoma peritonei (also sometimes called “low-grade mucinous carcinoma peritonei”).

The major considerations regarding mucinous lesions of the appendix are location, degree of peritoneal spread, and cytomorphology of the epithelium [1]. It is critical to address whether the lesion is localized in the appendix, whether it had invaded the appendiceal wall, extended beyond the appendiceal serosa but limited to the left lower quadrant, or whether it has spread to involve the entire peritoneal cavity [2]. Reportedly, the classification of these neoplasms historically has been challenging due to several factors. LAMNs localized in the appendix typically behave as benign neoplasms; however, as soon as neoplastic epithelium escapes the appendix, there is a significant rise in morbidity and mortality, even if cytology remains bland. Pseudomyxoma peritonei is clinically defined by the presence of intraperitoneal mucin, with or without associated mucin-producing epithelium; it can be progressive and frequently fatal [13]. The histologic grade of the peritoneal disease is considered a crucial reportable criterion. Pseudomyxoma peritonei with scant, low-grade epithelium has good prognosis and much more indolent clinical course, while pseudomyxoma peritonei with abundant high-grade epithelium has been found to have a much more aggressive clinical course [5]. Therefore, grading the epithelium in peritoneal disease is important for management and prognosis. However, even with low-grade disease, the most common complication is bowel obstruction. Clear communication and mutual understanding between a pathologist and a treating clinician are essential in establishing an accurate diagnosis and guiding treatment. Importantly, LAMN should be considered in the clinical differential diagnosis for mucinous vaginal discharge.

## References

[1] Panarelli N. C., Yantiss R. K. (2011). Mucinous neoplasms of the appendix and peritoneum. *Archives of Pathology & Laboratory Medicine*.

[2] Usera P. C., Ferguson D. C., Cragun J. M. (2013). Mucinous neoplasms of the appendix and ovary: confusing terminology and clinical impact. *American Journal of Clinical and Experimental Obstetrics and Gynecology*.

[3] McKenney J. K., Soslow R. A., Longacre T. A. (2008). Ovarian mature teratomas with mucinous epithelial neoplasms: morphologic heterogeneity and association with pseudomyxoma peritonei. *American Journal of Surgical Pathology*.

[4] Vang R., Gown A. M., Zhao C. (2007). Ovarian mucinous tumors associated with mature cystic teratomas: morphologic and immunohistochemical analysis identifies a subset of potential teratomatous origin that shares features of lower gastrointestinal tract mucinous tumors more commonly encountered as secondary tumors in the ovary. *American Journal of Surgical Pathology*.

[5] Pai R. K., Beck A. H., Norton J. A., Longacre T. A. (2009). Appendiceal mucinous neoplasms: clinicopathologic study of 116 cases with analysis of factors predicting recurrence. *American Journal of Surgical Pathology*.

[6] Tahan G., Koc I., Tetikkurt U. S., Yucel O. (2007). Mucinous tumor of uncertain malignant potential in a perforated appendectomy specimen. A case report. *Journal of Gastrointestinal and Liver Diseases*.

[7] Sieren L. M., Collins J. N., Weireter L. J. (2010). The incidence of benign and malignant neoplasia presenting as acute appendicitis. *The American Surgeon*.

[8] Yantiss R. K., Shia J., Klimstra D. S., Hahn H. P., Odze R. D., Misdraji J. (2009). Prognostic significance of localized extra-appendiceal mucin deposition in appendiceal mucinous neoplasms. *American Journal of Surgical Pathology*.

[9] Samet I., Cormier B., Mowlawi H., Philippe A., Arbion F., Fétissof F. (2009). Endometrial and endocervical lesions associated with pseudomyxoma peritonei: a case report. *Annales de Pathologie*.

[10] McVeigh G., Shah V., Longacre T. A., McCluggage W. G. (2015). Endometrial involvement in pseudomyxoma peritonei secondary to low-grade appendiceal mucinous neoplasm: report of 2 cases. *International Journal of Gynecological Pathology*.

[11] Shaw K. C., Kokh D., Ioffe O. B., Staats P. N. (2015). ‘Pseudomyxoma Endometrii’: endometrial deposition of acellular mucin from a low-grade appendiceal mucinous neoplasm as a rare mimic of myxoid uterine tumors. *International Journal of Gynecological Pathology*.

[12] Misdraji J., Yantiss R. K., Graeme-Cook F. M., Balis U. J., Young R. H. (2003). Appendiceal mucinous neoplasms: a clinicopathologic analysis of 107 cases. *American Journal of Surgical Pathology*.

[13] Misdraji J. (2010). Appendiceal mucinous neoplasms: controversial issues. *Archives of Pathology and Laboratory Medicine*.

